# Myocarditis, hepatitis, and pancreatitis in a patient with coxsackievirus A4 infection: a case report

**DOI:** 10.1186/1743-422X-11-3

**Published:** 2014-01-13

**Authors:** Nobuhiro Akuzawa, Naoyuki Harada, Takashi Hatori, Kunihiko Imai, Yonosuke Kitahara, Shinji Sakurai, Masahiko Kurabayashi

**Affiliations:** 1 Department of Internal Medicine, Social Insurance Gunma Chuo General Hospital, 1-7-13 Koun-cho, 371-0025 Maebashi, Gunma, Japan; 2 Department of Pathology, Social Insurance Gunma Chuo General Hospital, 1-7-13 Koun-cho, 371-0025 Maebashi, Gunma, Japan; 3 Department of Medicine and Biological Science, Gunma University Graduate School of Medicine, 3-39-22 Showa-machi, 371-8511 Maebashi, Gunma, Japan

**Keywords:** Coxsackievirus, Liver dysfunction, Myocarditis, Pancreatitis, Rectal cancer

## Abstract

Viral myocarditis presents with various symptoms, including fatal arrhythmia and cardiogenic shock, and may develop chronic myocarditis and dilated cardiomyopathy in some patients. We report here a case of viral myocarditis with liver dysfunction and pancreatitis. A 63-year-old man was admitted to our hospital with dyspnea. The initial investigation showed pulmonary congestion, complete atrioventricular block, left ventricular dysfunction, elevated serum troponin I, and elevated liver enzyme levels. He developed pancreatitis five days after admission. Further investigation revealed a high antibody titer against coxsackievirus A4. The patient’s left ventricular dysfunction, pancreatitis, and liver dysfunction had resolved by day 14, but his troponin I levels remained high, and an endomyocardial biopsy showed T-lymphocyte infiltration of the myocardium, confirming acute myocarditis. The patient underwent radical low anterior resection five weeks after admission for advanced rectal cancer found incidentally. His serum troponin I and plasma brain natriuretic peptide levels normalized six months after admission. He has now been followed-up for two years, and his left ventricular ejection fraction is stable.

This is the first report of an adult with myocarditis and pancreatitis attributed to coxsackievirus A4. Combined myocarditis and pancreatitis arising from coxsackievirus infection is rare. This patient’s clinical course suggests that changes in his immune response associated with his rectal cancer contributed to the amelioration of his viral myocarditis.

## Background

Myocarditis can present with a wide range of symptoms, ranging from mild dyspnea to chest pain, cardiogenic shock, and fatal arrhythmia. The main cause of myocarditis is current or recent viral infection [1]. Enteroviruses, specifically Coxsackievirus (CV) group B serotypes, have traditionally been perceived as the predominant viral cause [2], although adenoviruses, parvovirus B19, and human herpesvirus 6 can also cause myocarditis [3-5].

The pathophysiological progression of viral myocarditis includes three distinct phases [3]. The first phase is characterized by a nonspecific innate immune response, causing virus-mediated cell lysis and the indirect destruction of cardiomyocytes [3,6]. During the second phase, a virus-specific immune response, including CD8^+^ lymphocytes, acts to eliminate the causative viruses, leading to heart failure with the destruction of the infected cardiomyocytes [3]. In the third phase, commonly a few weeks after infection, the destroyed cardiomyocytes are replaced by diffuse fibrosis and progressive biventricular dilatation, resulting in cardiac failure [1,3]. Approximately 50% of patients with viral myocarditis develop chronic myocarditis, and 21% develop dilated cardiomyopathy (DCM) [7]. A longitudinal study reported that the immune clearance of viruses during or after the acute phase of myocarditis correlates with improvements in the left ventricular ejection fraction (LVEF) [8]. In chronic myocarditis or DCM, a persistent viral presence on endomyocardial biopsy (EMB) specimens is associated with an increased mortality rate [9]. However, viral genomic RNA and capsid protein are detectable in EMB specimens in no more than 35% and 10% of cases, respectively [6]. Therefore, the relationship between persistent viral infection and progression from acute myocarditis to chronic irreversible cardiomyopathy is unclear.

We present here a rare case of CVA4 infection causing acute myocarditis with concomitant pancreatitis and liver dysfunction. The patient’s antibody titers against CVA4 were significantly elevated during the recovery period, and a pathological examination of an EMB specimen showed interstitial infiltration with CD3-positive lymphocytes, despite a normal LVEF on left ventriculography, confirming acute myocarditis. His plasma brain natriuretic peptide (BNP) and serum troponin I (TnI) levels remained elevated. Five weeks after admission, the patient underwent radical low anterior resection for advanced rectal cancer, found by chance. Interestingly, his plasma BNP and serum TnI levels returned to normal after surgery. The patient’s clinical course suggests that the immune modulation associated with his rectal cancer and surgery favorably affected his acute myocarditis, preventing its progression to chronic myocarditis or DCM.

## Case report

A 63-year-old man was admitted to our hospital with a three-day history of dyspnea and fatigue, which had gradually increased until he experienced dyspnea and fatigue at rest. He had taken amlodipine (5 mg/day) for hypertension for the preceding five years, but had no other remarkable past history or family history. He was a nonsmoker and did not consume alcohol. Two weeks previously, he had presented with flu-like symptoms, including fever, sore throat, cough, and diarrhea, which had completely resolved within a week.

**Figure 1:**
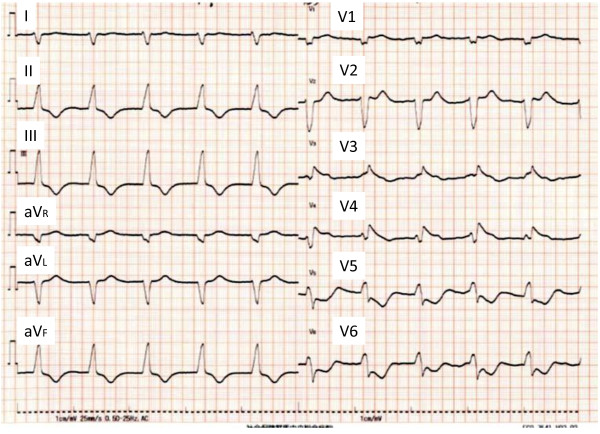
**Electrocardiography on admission.** Electrocardiography showed atrial fibrillation and an accelerated idioventricular rhythm (62 beats/min), suggesting complete atrioventricular block.

On admission, his height was 168.3 cm, weight 65.0 kg, and temperature 35.8°C. He was hypotensive, with a blood pressure of 72/52 mmHg, but his heart rate was not elevated (61 beats/min). A physical examination revealed cyanosis of the lips, distended external jugular veins, pretibial edema in both legs, coarse crackles over the lower bilateral lung fields, and mild enlargement of the liver. He was obviously short of breath in room air, with blood O_2_ saturation of 93%, partial O_2_ pressure of 61.7 mmHg, and partial CO_2_ pressure of 16.7 mmHg. A chest X-ray showed mild pulmonary congestion and right pleural effusion. Electrocardiography showed atrial fibrillation and an accelerated idioventricular rhythm, suggesting complete atrioventricular block (Figure 1). Transthoracic echocardiography showed moderately impaired left ventricular function with diffuse hypokinesis, but no pericardial effusion or enlargement of the right heart. The end-diastolic left ventricular dimension was 56 mm, the end-systolic left ventricular dimension was 44 mm, and LVEF calculated with the Teichholz formula was 42.9%. The interventricular septal thickness was 11 mm and the posterior left ventricular wall thickness was 12 mm. The diameter of the inferior vena cava was increased to 22 mm, with no respiratory variation. Color Doppler echocardiography showed mild tricuspid regurgitation and the estimated pressure gradient between the right atrium and right ventricle was 25.1 mmHg. Laboratory tests showed neutrophil-dominant leukocytosis, liver dysfunction, and renal insufficiency (Table 1). His serum creatine phosphokinase (CPK) MB isoenzyme, serum TnI, and plasma BNP levels were markedly elevated. Abdominal ultrasonography showed no abnormalities, other than moderately dilated hepatic veins. Coronary angiography was not performed because of the patient’s reduced renal function. The patient developed hemodynamically significant bradycardia (28 beats/min) soon after admission, and a temporary transvenous pacing wire was placed in the right ventricle.

**Table 1:** Laboratory findings on admission

***Hematology***		**Normal range**
White blood cells*	13700/mm^3^	3300 – 9000/mm^3^
Red blood cells	467 × 10^4^/mm^3^	430 – 570 × 10^4^/mm^3^ (Male)
Hemoglobin	13.6 g/dl	13.5 – 17.5 g/dl (Male)
Hematocrit	41.3%	39.7 – 52.4% (Male)
Platelets	29.3 × 10^4^/mm^3^	14.0 – 34.0 × 10^4^/mm^3^
***Differential white blood cell count***		
Segmented neutrophils*	70.0%	34.0 – 70.0%
Band cells, stab cells*	15.0%	1.0 – 7.0%
Eosinophils	1.0%	0 - 8.0%
Monocytes	5.0%	2.0 – 10.0%
Lymphocytes*	9.0%	18.0 – 49.0%
***Biochemistry***		
Total protein*	6.2 g/dl	6.7 – 8.3 g/dl
Albumin*	3.4 g/dl	3.8 – 5.2 g/dl
AST*	2660 IU/l	10 – 40 IU/l
ALT*	2037 IU/l	5 – 45 IU/l
LDH*	3307 IU/l	120 – 240 IU/l
ALP*	793 IU/l	100 – 325 IU/l
γ-GTP*	197 IU/l	10 – 50 IU/l
Total bilirubin*	1.7 mg/dl	0.2 – 1.2 mg/dl
Direct bilirubin*	1.0 mg/dl	0 – 0.4 mg/dl
CPK*	978 IU/l	60 – 270 IU/l
CPK-MB*	231 ng/ml	< 5.2 ng/ml
Blood urea nitrogen*	63.4 mg/dl	8.0 – 20.0 mg/dl
Creatinine*	3.01 mg/dl	0.61 - 1.04 mg/dl
Sodium	141 mEq/l	135 – 145 mEq/l
Potassium	3.8 mEq/l	3.5 – 5.0 mEq/l
Chloride	106 mEq/l	98 – 108 mEq/l
Calcium	8.4 mg/dl	8.4 – 10.4 mg/dl
Phosphorus	3.1 mg/dl	2.5 – 4.5 mg/dl
Glucose	99 mg/dl	70 – 109 mg/dl
C-reactive protein*	5.50 mg/dl	< 0.30 mg/dl
BNP*	4806 pg/ml	< 18.4 pg/ml
Troponin-I*	28.4 ng/ml	< 0.04 ng/ml
***Blood coagulation test***		
PT-INR*	2.19	0.85-1.15
***Serology***		
HBsAg	Negative	Negative
HCV-RNA	Negative	Negative
IgM anti-HAV Ab	Negative	Negative
Anti-HIV Ab	Negative	Negative

*These values are outside the normal ranges.

*Abbreviations:AST* aspartate aminotransferase, *ALT* alanine aminotransferase, *LDH* lactate dehydrogenase, *ALP* alkaline phosphatase, *γ-GTP* gamma-glutamyltranspeptidase, *CPK* creatine phosphokinase, *BNP* brain natriuretic peptide, *PT-INR* international normalized ratio of prothrombin time, *APTT* activated partial thromboplastin time.

The intravenous administration of dopamine (10 μg/kg/min), unfractionated heparin (15,000 units/day), and furosemide (20 mg/day) was begun on the day of admission. The patient’s systolic blood pressure increased to about 120 mmHg and stabilized. The low-dose intravenous administration of human atrial natriuretic peptide (0.025 μg/kg/min) was begun on day 2. Nonsustained monomorphic ventricular tachycardia lasting for about 1 min was observed several times on day 2, but had no significant hemodynamic impact, and was effectively treated with an intravenous bolus injection of amiodarone (150 mg). On day 3, the patient’s atrioventricular conduction returned to normal and his daily urine output increased to 3500 mL/day. On day 4, echocardiography showed an improvement in LVEF to 55.2% and the temporary pacing wire was removed. His serum CPK levels peaked on day 4 at 3229 IU/L. On day 7, a chest X-ray showed resolution of the right pleural effusion and his serum creatinine level returned to normal (0.98 mg/dL). The intravenous administration of human atrial natriuretic peptide was stopped, and the patient commenced a regimen of oral losartan (25 mg/day), spironolactone (12.5 mg/day), azosemide (30 mg/day), and warfarin (3.0 mg/day). Supplemental oxygen was no longer needed.

**Figure 2:**
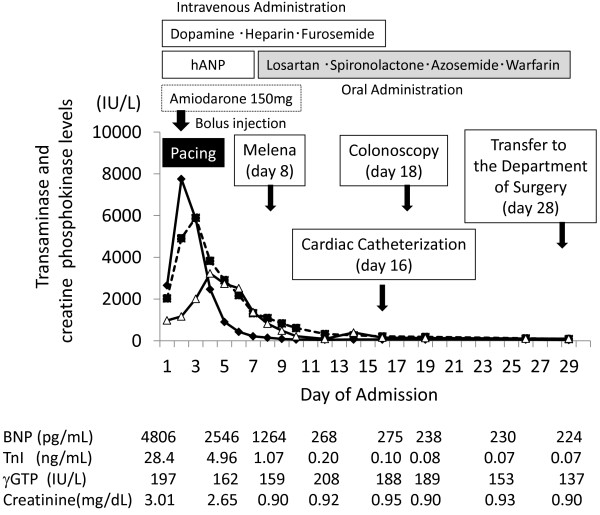
**Clinical course of the patient after admission.** Levels of serum aspartate aminotransferase (AST; diamonds), alanine aminotransferase (ALT; squares), and creatinine phosphokinase (CPK; triangles) were high. The AST level peaked on day 2, ALT on day 3, and CPK on day 4. The levels of brain natriuretic peptide (BNP) and troponin I (TnI) were highest at admission and subsequently decreased, but did not return to normal. The gamma-glutamyl transpeptidase (*γ*-GTP) level was moderately elevated during hospitalization. The serum creatinine level returned to normal by day 7.

**Figure 3:**
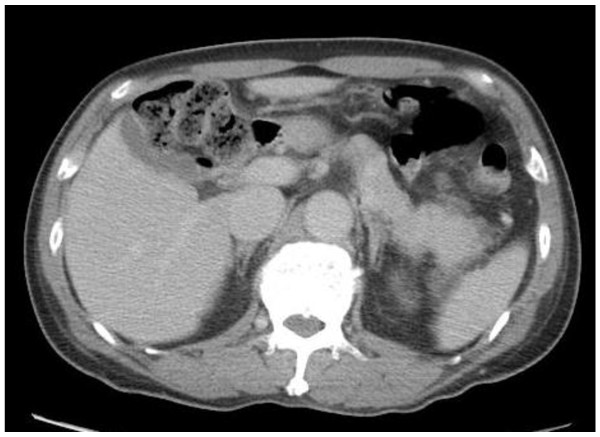
**Abdominal computed tomography (CT) on day 5.** Abdominal CT to investigate the cause of epigastric pain showed localized swelling of the pancreatic body and tail, with no cholelithiasis or tumor obstructing the common bile duct or pancreatic duct, suggesting idiopathic pancreatitis.

The patient’s liver dysfunction improved after admission. His aspartate aminotransferase (AST) level peaked on day 2 (7752 IU/L) and his alanine aminotransferase (ALT) level peaked on day 3 (5899 IU/L) (Figure 2). His biliary enzyme levels and total bilirubin also increased, but not as much as his AST and ALT levels. His prolonged prothrombin time returned to normal on day 7. However, he complained of epigastric pain on day 5, and laboratory tests showed increased serum and urine amylase levels. Abdominal computed tomography showed swelling of the pancreatic body and tail, with increased retroperitoneal adipose-tissue density, suggesting pancreatitis (Figure 3). No cholelithiasis or tumor occluding the common bile duct or pancreatic duct was observed, and his plasma immunoglobulin G4 level was normal. We administered intravenous gabexate mesilate (600 mg/day) and ceftriaxone (2 g/day) from day 5 to day 12. The patient’s abdominal symptoms resolved after a few days, and he recovered without complications. A small amount of melena was observed on day 8, and a total colonoscopy was scheduled. Both his liver dysfunction and pancreatitis had resolved by day 14.

**Figure 4:**
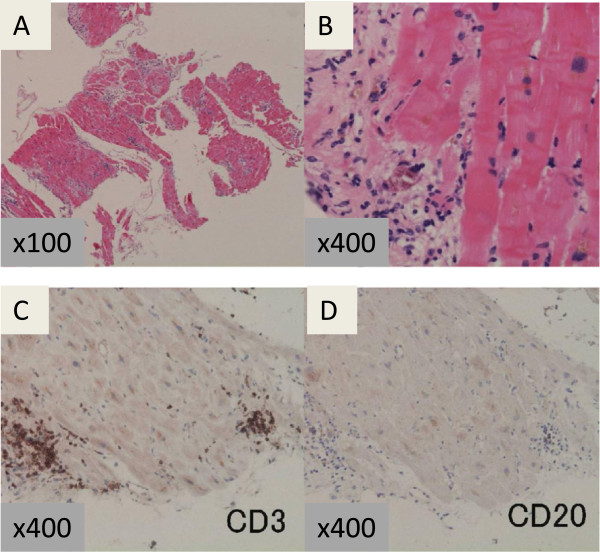
**Histopathological examination of a biopsy specimen from the left ventricle.** Low-power view (× 100) **(A)** and high-power view (× 400) **(B)** of the biopsy specimen obtained from the left ventricle, stained with hematoxylin and eosin, showing lymphocytic infiltration into the interstitium and mild necrosis of the myocardium. Immunohistochemical staining with anti-CD3 **(C)** and anti-CD20 antibodies **(D)**. CD3-positive lymphocytes were predominant.

Cardiac catheterization on day 16 showed normal pressure data and a normal cardiac index (3.2 L/min/m^2^). Coronary angiography and left ventriculography showed no abnormalities and normal LVEF (64.2%). A pathological examination of an EMB specimen of the left ventricle showed necrotic cardiomyocytes adjacent to areas with interstitial infiltration of CD3-positive lymphocytes (Figure 4A–D), confirming acute myocarditis. The antibody titer against CVA4 increased from 1:4 on day 1 to 1:256 on day 15. On day 19, his plasma BNP level was 238 pg/mL and his serum TnI level was 0.08 ng/mL, and both these values continued to be slightly elevated thereafter.

A colonoscopy on day 18 showed a protruding rectal lesion above the level of the peritoneal reflection. The plasma carcinoembryonic antigen level was 4.7 ng/mL (normal: 0–4.9 ng/mL) and the cancer antigen 19–9 level was 434.6 U/mL (normal: 0–36.9 U/mL). A pathological examination of a biopsy specimen of the rectal lesion showed a moderately differentiated tubular adenocarcinoma invading the muscularis propria. Transesophageal echocardiography on day 27 showed no signs of left atrial thrombus, and the administration of warfarin was stopped in preparation for surgery. The patient was transferred to the Department of Surgery on day 28, and underwent a low anterior resection on day 35. The postoperative diagnosis was stage II rectal cancer (T3N0M0, Union for International Cancer Control Classification). His postoperative recovery was uneventful, with no complications such as heart failure or arrhythmia. He was discharged on day 45. A histological examination of the surgical specimen showed lymphovascular invasion, and adjuvant chemotherapy with folinic acid, fluorouracil, and oxaliplatin was commenced a month after discharge. Interestingly, his plasma BNP level decreased to less than half the preoperative value (78 pg/mL) and his serum TnI level returned to normal (< 0.04 ng/mL) three weeks after surgery. His plasma BNP level returned to normal (18 pg/mL) four months after surgery. Oral medications for heart failure were stopped five months after discharge. The patient underwent a partial hepatectomy six months after discharge for a solitary metastasis in the liver, but his myocarditis did not recur. Adjuvant chemotherapy was continued until 12 months after surgery. The patient has now been followed-up for two years, with stable LVEF and no recurrence of myocarditis or rectal cancer.

## Discussion

Coxsackieviruses belong to the genus *Enterovirus*, in the family *Picornaviridae*, and are important viral pathogens, causing myocarditis and pancreatitis. CVs are subdivided according to their pathogenicity in suckling mice aged less than 48 h into CVA, which causes flaccid paralysis, and CVB, which causes spastic paralysis [10-12]. There are 23 CVA serotypes and six CVB serotypes [12]. Although CVs are known to cause myocarditis, most cases are attributed to CVB serotypes [6]. CVA infection can cause viral myocarditis, but it has a more benign course than CVB myocarditis [12]. Serotypes A4 and A16 have been most frequently implicated in the rare cases of CVA-associated myocarditis reported [13]. Combined myocarditis and pancreatitis arising from CV infection is very rare, with only three previously reported cases, which were all attributed to CVB infection [14,15]. Ischemic pancreatitis was unlikely in our patient because of the late disease onset (day 5) and hemodynamic stability after admission. Moreover, although we did not stop any medications, including diuretics, the patient’s pancreatitis improved spontaneously. Therefore, it is more likely that his pancreatitis developed from his CV infection. To our knowledge, this is the first reported case of an adult patient with myocarditis and pancreatitis attributable to CVA infection. It is unclear why combined myocarditis and pancreatitis is uncommon in CV-infected humans. In a mouse model, the viral titer was higher in the pancreas than in the heart, liver, or spleen during the early stage of CVB infection [16]. In humans, CVB3 capsid protein VP1 was detected in both the cardiomyocytes and islets of endocrine cells in patients who died of acute CVB myocarditis [17]. These results suggest that the rate of latent or asymptomatic pancreatitis may be higher than previously thought in patients with myocarditis attributable to CV infection.

The course of our patient’s myocarditis is interesting. Although his LVEF on echocardiography improved soon after admission and normalized on left ventriculography by day 16, an EMB specimen on the same day still showed prominent T-lymphocyte infiltration with a slightly elevated serum TnI level. His serum TnI level had returned to normal three weeks after surgery, and his plasma BNP level had also decreased at this time. This suggests that resection of the rectal cancer may have resulted in the improvement of his myocarditis and inhibited any progression to chronic myocarditis or DCM. The patient’s normal LVEF during the two-year follow-up supports this conjecture. The relationship between persistent viral infection and the establishment of chronic myocarditis is controversial. A recent study reported that regulatory T cells (Tregs), which are positive for CD4, CD25, and Foxp3, reduced viral titers and suppress cardiac damage in CVB-infected mice [18]. Furthermore, the myocardial Treg frequency was reduced during the course of CVB3 myocarditis in BALB/c mice and the adoptive transfer of Tregs resulted in the significant amelioration of cardiac fibrosis [19]. Tregs maintain immunohomeostasis and limit autoimmunity, but there may be high levels of Tregs in the blood and tumor tissues of cancer patients, leading to the suppression of antitumor immunity [20]. The proportion of Tregs is significantly higher in colon cancer patients than in healthy controls, and decreases significantly after the radical resection of the tumors [21]. Increased Treg activity also suppresses CD8^+^ T-cell responses to specific antigens of various viruses [22]. The limitations of our report include our inability to determine the myocardial frequency or activity of Tregs before and after surgery, but our findings suggest that the modulation of the immune response associated with rectal cancer and surgery, including a change in Treg activity, may have contributed to the amelioration of myocarditis in our patient. More specifically, the increased Treg activity associated with rectal cancer may have had an inhibitory effect on his cardiac fibrosis and CD8^+^ T-cell-associated myocardial damage during the acute or subacute period of myocarditis, leading to the benign clinical course of this patient, although the onset of myocarditis per se could not be prevented. However, a point of uncertainty persists because reduced Treg activity after surgery might have exacerbated his myocarditis. Despite this, the patient’s myocarditis improved. This suggests that components other than Tregs were involved in the improvement in his myocarditis, such as mesenchymal stromal cells (MSCs), which have been identified in the bone marrow and have unique immunoregulatory and regeneration properties [23]. MSCs reduced myocardial inflammation by reducing the expression of proinflammatory cytokines and inducing an increase in Tregs and CD4^+^- and CD8^+^-T-cell apoptosis in CVB3-infected mice [24]. However, it should be noted that no Treg or MSC activity has yet been reported in patients with chronic myocarditis or DCM. Although our patient’s clinical course after surgery included the resolution of his myocarditis, further research is required to clarify the mechanisms involved.

## Conclusion

We have presented a rare case of myocarditis, liver dysfunction, and pancreatitis arising from CVA4 infection in a patient with undiagnosed advanced rectal cancer. His clinical course was good and his elevated serum TnI and plasma BNP levels returned to normal over four months after the radical resection of his rectal cancer. These features suggest that changes in the immune response to myocarditis that were associated with the patient’s rectal cancer may have had a favorable effect on his myocarditis, thereby preventing the progression of acute myocarditis to chronic cardiomyopathy.

## Consent

Written informed consent was obtained from the patient for publication of this case report and any accompanying images. A copy of this written consent is available for review by the Editor-in-Chief of this journal.

## Abbreviations

CV: Coxsackievirus; EMB: Endomyocardial biopsy; LVEF: Left ventricular ejection fraction; BNP: Brain natriuretic peptide; TnI: Troponin I; CPK: Creatine phosphokinase; AST: Aspartate aminotransferase; ALT: Alanine aminotransferase; DCM: Dilated cardiomyopathy; MSC: Mesenchymal stromal cell; Treg: Regulatory T cell.

## Competing interests

The authors declare that they have no competing interests.

## Authors’ contributions

NA drafted the manuscript. NH and TH collected the patient data and monitored the patient throughout the whole follow-up period. KH, YK, and SS edited the manuscript. MK participated in the study design and coordination and helped to draft the manuscript. All authors have read and approved the final manuscript.
